# Epidemiology of foodborne Norovirus outbreaks in Catalonia, Spain

**DOI:** 10.1186/1471-2334-8-47

**Published:** 2008-04-14

**Authors:** Ana Martinez, Angela Dominguez, Nuria Torner, Laura Ruiz, Neus Camps, Irene Barrabeig, Cesar Arias, Josep Alvarez, Pere Godoy, Pilar Jorgina Balaña, Analia Pumares, Rosa Bartolome, Dolors Ferrer, Unai Perez, Rosa Pinto, Javier Buesa, The Catalan Viral Gastroenteritis Study Group

**Affiliations:** 1 Department of Health, Autonomous Government of Catalonia, Barcelona, Spain; 2 Department of Public Health, University of Barcelona, Spain; 3 CIBER Epidemiology and Public Health (CIBERESP), Spain; 4 Department of Microbiology, Hospital Universitari Vall d'Hebron, Barcelona, Spain; 5 Public Health Laboratory, Public Health Agency of Barcelona, Spain; 6 Enteric Virus Laboratory, University of Barcelona, Spain; 7 Microbiology Department, University of Valencia, Spain

## Abstract

**Background:**

Noroviruses are one of the principal biological agents associated with the consumption of contaminated food. The objective of this study was to analyse the size and epidemiological characteristics of foodborne outbreaks of gastroenteritis in Catalonia, a region in the northeast of Spain.

**Methods:**

In all reported outbreaks of gastroenteritis associated with food consumption, faecal samples of persons affected were analysed for bacteria and viruses and selectively for parasites. Study variables included the setting, the number of people exposed, age, sex, clinical signs and hospital admissions. The study was carried out from October 2004 to October 2005.

**Results:**

Of the 181 outbreaks reported during the study period, 72 were caused by *Salmonella *and 30 by norovirus (NoV); the incidence rates were 14.5 and 9.9 per 100,000 person-years, respectively. In 50% of the NoV outbreaks and 27% of the bacterial outbreaks (p = 0.03) the number of persons affected was ≥10; 66.7% of NoV outbreaks occurred in restaurants; no differences in the attack rates were observed according to the etiology. Hospitalizations were more common (p = 0.03) in bacterial outbreaks (8.6%) than in NoV outbreaks (0.15%). Secondary cases accounted for 4% of cases in NoV outbreaks compared with 0.3% of cases in bacterial outbreaks (p < 0.001)

**Conclusion:**

Norovirus outbreaks were larger but less frequent than bacterial outbreaks, suggesting that underreporting is greater for NoV outbreaks. Food handlers should receive training on the transmission of infections in diverse situations. Very strict control measures on handwashing and environmental disinfection should be adopted in closed or partially-closed institutions.

## Background

Diseases resulting from the consumption of contaminated food cause a considerable disease burden in developed countries [1], and thus it is important to determine their etiology and food vehicles. Although there are difficulties in associating a specific food with the appearance of cases or outbreaks of gastroenteritis [2], reports agree that noroviruses (NoV) (formerly Norwalk-like viruses) are one of the foremost biological agents involved in cases of gastroenteritis associated with food consumption [3].

The stability of NoV in various environmental conditions means that they can remain infectious in frozen and refrigerated food and even in food heated to 60°C for 30 minutes [4], which explains why they can be easily transmitted by foods contaminated by contact with human faecal matter or by unhygienic food handling [5].

The infective dose of NoV is very low: new infections may be produced by person-to-person transmission of very small amounts of virus. Therefore, secondary cases usually appear in foodborne outbreaks caused by a single exposure [6].

The available evidence on foodborne gastroenteritis outbreaks due to NoV is based on national and international public health activities [6,7]. Although most laboratories are equipped to analyse bacterial processes, few are able to make a diagnosis of viral causes of gastroenteritis and, therefore, confirmation of a possible viral cause of gastroenteritis is not always sought [8].

Analysis of the official statistics provided by different health authorities is frequently partial and their interpretation is complex [9].

Studies of the epidemiology of foodborne NoV outbreaks in each community are necessary, even though they represent only a part of the real situation due to clinical and epidemiological underreporting and laboratory difficulties. Knowledge of outbreaks and the distribution of specific strains is also necessary to carry out interventions at a local level that allow the prevention of new outbreaks [6,10].

The objective of this study was to determine the size and epidemiological characteristics of foodborne outbreaks due to NoV in Catalonia between October 2004 and October 2005 and compare them with bacterial outbreaks.

## Methods

We carried out a prospective study of foodborne outbreaks occurring between 15 October 2004 and 30 October 2005 in Catalonia, a region in the northeast of Spain, with a population of 6.9 million.

A foodborne outbreak was defined as two or more cases with similar symptoms resulting from the ingestion of a common food when this was confirmed by epidemiological and/or microbiological analysis.

When an outbreak was reported to public health authorities, a routine investigation was carried out to determine the characteristics of the cases and the possible food involved using a standardized questionnaire. Likewise, clinical and food samples were collected for laboratory analysis to identify the causal agent. In addition to standard microbiological tests to rule out bacterial and parasitic causes, enzyme linked immunosorbent assay and RT-PCR techniques were carried out on faecal samples of cases, and of food handlers when the outbreak was not limited to the family setting, to detect viruses.

Stool samples were plated on selective and differential media to study *Salmonella *(MacConkey agar, Salmonella-Shigella agar, Xylose-Lysine-Desoxycholate agar and Selenite enrichment broth), *Shigella *(MacConkey agar and Salmonella-Shigella agar), Shiga toxin-producing strains of O157:H7 *Escherichia coli *(MacConkey agar with sorbitol), *Yersinia *(Cefsulodin-Irgasan-Novobiocin, CIN agar), *Campylobacter *(Charcoal agar), *Vibrio *(Thiosulfate Citrate Bile salt Sucrose, TCBS agar) and *Aeromonas *spp (Pseudomonas-Aeromonas agar with 100,000 IU per litre of Penicillin G, GSP agar).

In outbreaks where a parasitic infection was suspected, the diagnosis was established by direct microscopic examination or after concentration of preserved stool (Merthiolate-iodine-formalin and 10% formalin) to determine the presence of ova, trophozoites or cysts. *Cryptosporidium *oocysts were examined by stained fecal materials (Auramine and Ziehl-Neelsen stains).

Enzyme immunoassays for NoV genogroups I and II (IDEIA™ NoV, DakoCytomation), rotavirus group A (IDV Rotavirus-96.Izasa), astrovirus (IDEIA™ Astrovirus, DakoCytomation) and adenovirus serotypes 40 and 41 (IDV Adenovirus-96.Izasa) and RT-PCR were performed.

For NoV, RT-PCR primers designed for partial RNA polymerase region (ORF1) were used: NVp110 (5'-ACD ATY TCA TCA TCA CCA TA-3') for RT and JV12 (5'-ATA CCA CTA TGA TGC AGA TTA-3'), and JV13 (5'-TCA TCA TCA CCA TGA AAA GAC-3')for PCR [11]. For rotavirus, the primers used were VP6-3 (5'-GCT TTA AAA CGA AGT CTT CAA C-3') and VP6-4 (5'-GGT AAA TTA CCA ATT CCT CCA G-3') [12]. The primers used for adenovirus were hexAA1885 (5'-GCCGCAGTGGTCTTACATGCACATC-3') and hexAA1913 (5'-CAGCACGCCGCGGATGTCAAAGT-3'), which amplify a 301 bp fragment within the hexon region of the adenovirus genome [13]. For the genogroup A astrovirus, primer set A1 (5'-CCTGCCCCGAGAACAACCAAGC-3') and A2 (5'-GTAAGATTCCCAGATTGGTGC-3') from the hypervariable region of the ORF1a of the astrovirus genome was used [14] and for the detection of genogroup B astrovirus, primer set A1bis (5'-CCTGCCCCCCGTATAATTAAAC-3') and A2bis (5'-ATAGGACTCCCATATAGGTGC-3') [15]. PCR products were detected in a 2% ethidium bromide-stained agarose gel and purified with the QIAquick PCR Purification Kit (QIAGEN, Hilden, Germany).

Norovirus genotyping systems and an automated sequencer (Applied Biosystems) was performed by sequencing the amplimers with the JV12 and JV13 primers using the ABI PRISM Big Dye Terminator Cycle Sequencing kit (Applied BiosBI PRISM 3700) [16]. Likewise, foods suspected of being involved in the outbreak were analysed when some remained.

An outbreak was considered as being caused by NoV if one or more samples were PCR positive for NoV or if cases fulfilled Kaplan's criteria [17].

The variables analysed included the agent, setting of the outbreak, date of appearance of the first case, number of people exposed, number of cases, age, sex, symptoms and hospitalization. In non-familial outbreaks, it was determined whether the case was primary or secondary. A secondary case was defined as someone who had not consumed the suspected food and in whom the onset of symptoms occurred after the maximum incubation period of the causal agent.

### Statistical Analysis

Differences between medians were compared using the Mann-Whitney U test. Differences between proportions were compared using the Χ^2 ^test or Fisher's exact test. The tests were two-tailed. An alpha level = 0.05 was considered statistically significant. Incidence rates and their 95% confidence intervals (CI) were calculated using the 2005 voter's list, assuming a Poisson distribution. Only outbreaks occurring in a natural year (15 October 2004 – 14 October 2005) were considered for the seasonal distribution and calculation of the incidence rates.

## Results

In the study period there were 181 foodborne gastroenteritis outbreaks due to all causes, of which *Salmonella *(72 outbreaks, 40%) and NoV (30 outbreaks, 17%) were the most-frequent (Table 1). Of the 30 foodborne NoV outbreaks, 20 (66.7%) occurred in restaurants, 6 (20%) in families, 2 (6.7%) in residential nursing homes, 1 (3.3%) in a school and 1 in a summer camp (3.3%). The number of samples analyzed was 1 to 3 in 10 outbreaks, 4 to 9 in 11 outbreaks and 10 or more in 8 outbreaks. There was only one NoV outbreak with no samples. NoV was identified as the sole agent in one or more samples from cases in 22 outbreaks and there was a mixed etiology in 3 outbreaks (with adenovirus, *Salmonella *and *Vibrio parahaemolyticus*, respectively); in the other 5 outbreaks, Kaplan's criteria were fulfilled. The genotype was determined In 10 outbreaks; 8 were genotype GGII.4 (Bristol/1993/UK) and 2 were GGII.2 (Melksham/1994/UK).

**Table 1:** Distribution of foodborne outbreaks according to etiology. Catalonia, 15 October 2004 – 30 October 2005

**Etiology**	**Number of outbreaks (%)**	**Number of persons affected (%)**
*Salmonella*	72 (39.8)	605 (29.4)
NoV	30 (16.6)	741 (35.0)
Other bacteria *	14 (7.7)	413 (20.1)
Vegetable toxins	16 (8.8)	57 2.7)
Other toxic substances	12 (6.6)	39 (1.9)
Unknown	37 (20.5)	263 (12.8)
**Total**	**181 (100.0)**	**2118 (100.0)**

* Staphylococcus aureus (6), *Clostridium perfringens *(6), *Campylobacter jejuni *(1), *Streptococcus pyogenes *(1).

A total of 741 people were affected in the 30 NoV outbreaks and 1018 in 86 bacterial outbreaks. The incidence rates per 100,000 person-years of the cases associated with outbreaks were 9.9 (95% CI 9.2–10.7) and 14.5 (95% CI 13.6–15.4), respectively.

Although all cases were primary in most of the non-family outbreaks, in 7 NoV outbreaks a total of 27 secondary cases were recorded (3.8%); in bacterial outbreaks only 2 secondary cases were detected (0.3%), both in the same outbreak (Table 2). The median time from the onset of the outbreaks until reporting of secondary cases was 48–72 hours.

**Table 2:** Distribution of primary and secondary cases in the NoV and bacterial foodborne outbreaks according to setting.* Catalonia, 15 October 2004 – 30 October 2005

	**NoV outbreaks**			**Bacterial outbreaks**		
	
**Setting**	**Primary cases**	**Secondary cases**	Total *	**Primary cases**	**Secondary cases**	Total *
Restaurants	499	6	505	298	-	298
Residential nursing homes	7	5	12	7	-	7
Hospitals	-	-	-	25	2	27
Schools	129	14	143	306	-	306
Summer camps	38	2	40	-	-	-
Cake shops	-	-	-	20	-	20
All outbreaks	673	27	700	656	2	658

*Family outbreaks excluded

The median age of cases was ≤15 years in 10% of NoV outbreaks and 6% of bacterial outbreaks, 16–59 years in 83% and 87%, respectively and ≥60 years in 7% in both groups. There were no significant differences between the gender distribution of NoV outbreaks (51.2% male and 48.8% female) and bacterial outbreaks (52.5% male and 47.5% female). The most frequent symptoms are shown in Table 3.

**Table 3:** Clinical characteristics of NoV and bacterial outbreaks. Catalonia, 15 October 2004 – 30 October 2005

	**NoV outbreaks**	**Bacterial outbreaks**	**p value**
No. outbreaks	30	86	
No. cases	741	1018	
Size of outbreak (median and range)	8.5 (2–174)	5.0 (2–123)	0.029^a^
Attack rate (median)	0.66	0.70	0.091^a^
Abdominal pain (%)	81.5	79.6	0.34 ^b^
Vomiting (%)	59.0	39.0	< 0.001 ^b^
Diarrhea (%)	68.5	87.0	< 0.001 ^b^
Nausea (%)	62.2	41.2	< 0.001 ^b^
Fever (%)	42.0	52.4	< 0.001 ^b^
Hospitalizations (%)	0.15	8.6	< 0.001 ^b^

^a ^Mann-Whitney U; ^b ^χ^2^

The size of the outbreaks ranged between 2 and 174 in NoV outbreaks and between 2 and 123 in bacterial outbreaks. In 50% of the NoV outbreaks and 27% of the bacterial outbreaks (p = 0.03) the number of cases was ≥10. In 70% of the NoV outbreaks and 74% of the bacterial outbreaks the attack rate was >50%. Hospitalization occurred in one case in the viral outbreaks (0.15%) and in 87 cases in bacterial outbreaks (8.6%), with the difference being statistically significant (p < 0.001) (Table 3).

**Figure 1:**
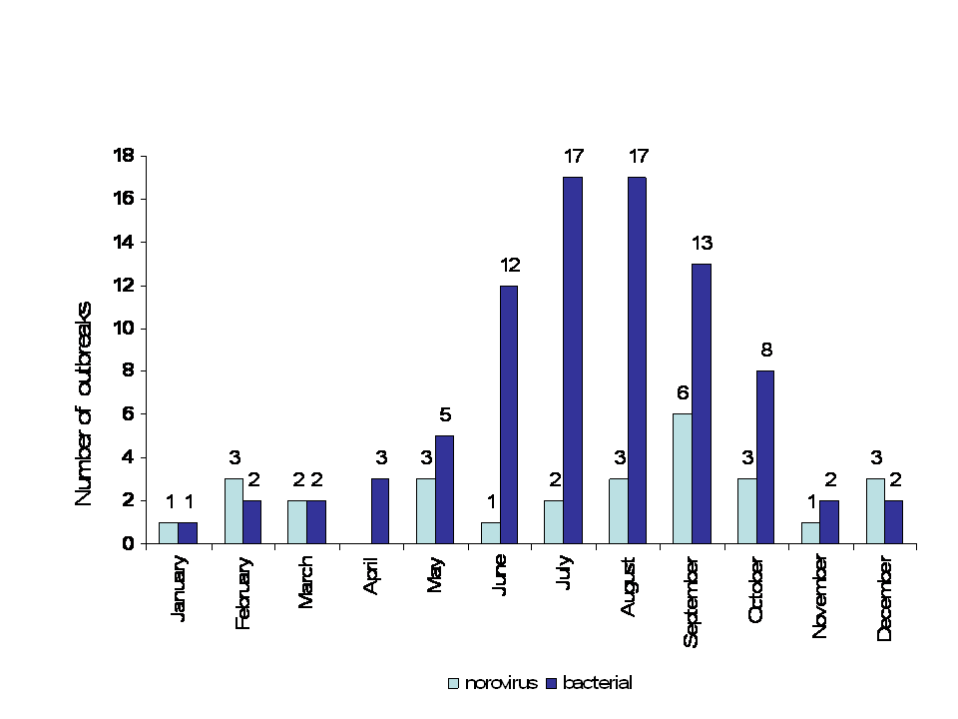
Monthly distribution of foodborne outbreaks. 15 October 2004 – 14 October 2005.

No seasonal pattern was observed in NoV outbreaks, while bacterial outbreaks showed an increase between June and October (Figure 1).

In the 17 NoV outbreaks in which the food vehicle was determined by epidemiological analysis, the most common vehicles were fish, and more specifically, bivalve molluscs (8 outbreaks, 26.7%), pastries (3 outbreaks, 10%) and vegetables (2 outbreaks; 6.6%); in the bacterial outbreaks, these figures were 2.3%, 3.5% and 0%, respectively, with only the differences for fish and vegetables being statistically significant. Foods purchased in cake shops were more frequently involved in NoV outbreaks (10%) than in bacterial outbreaks (3.5%) although the differences were not statistically significant (Table 4). Laboratory analysis of foods was possible in 9 of the 30 NoV outbreaks, although the virus was not detected in any outbreak; in 11 of the 86 bacterial outbreaks, the causal agent in the food was confirmed.

**Table 4:** Distribution of foods involved in NoV and bacterial outbreaks. Catalonia, 15 October 2004 – 30 October 2005

	**NoV outbreaks**	**Bacterial outbreaks**	p value *
Mayonnaise and similar	0	27 (31.4)	0.005
Other products containing egg	2 (6.7)	19 (22.1)	0.51
Fish and seafood	8 (26.7)	2 (2.3)	< 0.0001
Meat/sausage	1 (3.3)	6 (7.0)	1.0
Vegetables	2 (6.6)	0	0.04
Fowl	0	3 (3.5)	1.0
Cake shops	3 (10.0)	3 (3.5)	0.06
Others	1 (3.3)	9 (10.5)	0.67
Unknown	13 (43.3)	17 (19.8)	0.003

*Fisher test calculated comparing each food with respect to all foods involved

Stool samples from food handlers were analysed in 23 of 30 NoV outbreaks (76.7%) and one or more samples were positive in 17. The possible involvement of food handlers was detected by investigation in 13 outbreaks (43%) but was confirmed by molecular epidemiology in only one outbreak. Faecal samples from food handlers were analyzed in 30 of 86 bacterial outbreaks (34.9%) and the investigation confirmed the implication of a food handler as the source in 7 outbreaks (8.1%) by microbiology (matching types).

## Discussion

The results of this study emphasize the importance of foodborne transmission in gastroenteritis outbreaks due to NoV.

In this study, NoV was the second etiologic agent (30 outbreaks), only preceded by *Salmonella *(72 outbreaks). This is in agreement with other reports [8,18], although some studies have found NoV to be the first cause of foodborne outbreaks [3,19-21]. The incidence rate of the NoV cases associated with outbreaks was 9.9 per 100,000 person-years, less than the 15.6 found by Lindqvist et al in Sweden [21].

Widdowson et al [18] found that 25% of bacteria-negative outbreaks were not analyzed to detect viral causes, but this type of information is not normally available. In this study, all reported foodborne outbreaks were studied, searching first for bacteria and selectively for parasites and, if these were negative, for viruses. NoV outbreaks involved more cases, but less febrile cases and hospitalizations, showing that NoV outbreaks were less severe than bacterial outbreaks.

Although reported viral outbreaks were larger than bacterial outbreaks, the attack rates were similar, suggesting that smaller viral outbreaks are not reported. Cowden et al [22] in England found that underreporting of NoV was one hundred times greater than for *Salmonella *and 30 times greater than for *Campylobacter*.

In contrast with bacterial outbreaks, no seasonality was observed in NoV outbreaks. Some reports have found no seasonality in regard to NoV infection [22,23], while others have found an increase in winter in outbreaks involving person-to-person transmission [10], but not in foodborne outbreaks [24].

In this study, the food implicated was identified in only 57% of NoV outbreaks compared with 80% of bacterial outbreaks. In the United States, a 2002 study found levels of 47% and 76%, respectively [19], but other studies show results similar to ours [18]. With the exception of bivalve molluscs, laboratory techniques to detect NoV in foods still have a very low sensitivity [20,25].

The involvement of a food handler was suspected in 43% of NoV outbreaks, although the involvement was identified by molecular epidemiology in only one outbreak. Faecal samples should be routinely collected from patients and food handlers involved in the preparation of the foods consumed in order to demonstrate their involvement.

In our study, 27 of the 715 (3.8%) people affected by NoV in non-family outbreaks were secondary cases, a ten-fold greater proportion than in bacterial outbreaks. Some reports of foodborne NoV outbreaks mention secondary cases [25-27], but their frequency is not clear [6,27,28]. As the incubation period of NoV infections is very short, cases may occur from contact with an infected person rather than from consumption of the food and, therefore, the number of secondary cases detected should be considered inferior to the real number. Research into whether cases in foodborne outbreaks are primary or secondary should be enhanced.

Most secondary cases detected corresponded to residential nursing homes (45%) and schools (10%), where close contact is the norm. It is established that up to 30% of infected people continue to shed the virus for three weeks [29]. Therefore, when a gastroenteritis outbreak of viral etiology is suspected, strict measures with respect to handwashing and disinfection of surfaces should immediately be adopted and compliance checked [30-32].

The main limitations of this study were the small number of samples available to diagnose the outbreaks and the passive nature of the reporting on which the study was based.

Although the means for laboratory diagnosis are available, it is not always possible to obtain the minimum four positive samples necessary to attribute the outbreak to NoV [31,33,34]. In this study we considered that one sample positive for NoV was sufficient if tests for bacteria and parasites were negative and the clinical signs and epidemiology were compatible [17]. A recent study [35] shows that the number of samples is less important for NoV outbreak diagnosis when RT-PCR techniques are used (as in the present study) than when only ELISA techniques are used.

The passive surveillance system used during the study period may have resulted in less outbreaks being studied than really occurred. However, it is unlikely that this influenced the comparison between viral and bacterial outbreaks since, once the reports were received, the activities carried out were the same.

## Conclusion

In this study, NoV was the second causal agent of foodborne outbreaks after *Salmonella *and NoV outbreaks were larger than bacterial outbreaks, suggesting greater underreporting and, consequently, draw-backs in the investigation of NoV outbreaks. Given that the NoV has a human reservoir, a very low infective dose and prolonged persistence in the environment, food handlers should be aware of how they can transmit the infection in different situations and receive appropriate preventive training [5]. In order to avoid secondary cases, when a foodborne outbreak of viral gastroenteritis in closed or partially-closed institutions is suspected, rapid control measures should be adopted, with an emphasis on handwashing and correct disinfection of environmental surfaces [32].

## Abbreviations

NoV: Norovirus

RT-PCR: Reverse transcription polymerase chain reaction

CI: Confidence Interval

## Competing interests

The author(s) declare that they have no competing interests.

## Authors' contributions

AD and AM designed the study and drafted the manuscript. NT participated in the design and coordination and helped to draft the manuscript. LR performed the statistical analysis. RB, UP, RP, DF, and JB performed the microbiological analysis, IB, NC, JA, CA, PG, JPB, AP participated in the acquisition of outbreak data. All authors read and approved the final manuscript.

## Pre-publication history

The pre-publication history for this paper can be accessed here:


